# “Core” RxLR effectors in phytopathogenic oomycetes: A promising way to breeding for durable resistance in plants?

**DOI:** 10.1080/21505594.2021.1948277

**Published:** 2021-07-25

**Authors:** Jane Chepsergon, Thabiso E. Motaung, Lucy Novungayo Moleleki

**Affiliations:** Department of Biochemistry, Genetics and Microbiology, Forestry and Agricultural Biotechnology Institute, University of Pretoria, Pretoria, Gauteng, South Africa

**Keywords:** Oomycetes, durable-resistance, virulence, “core” RxLR effectors (CRE), *phytophthora* spp

## Abstract

Phytopathogenic oomycetes are known to successfully infect their hosts due to their ability to secrete effector proteins. Of interest to many researchers are effectors with the N-terminal RxLR motif (Arginine-any amino acid-Leucine-Arginine). Owing to advances in genome sequencing, we can now comprehend the high level of diversity among oomycete effectors, and similarly, their conservation within and among species referred to here as “core” RxLR effectors (CREs). Currently, there is a considerable number of CREs that have been identified in oomycetes. Functional characterization of these CREs propose their virulence role with the potential of targeting central cellular processes that are conserved across diverse plant species. We reason that effectors that are highly conserved and recognized by the host, could be harnessed in engineering plants for durable as well as broad-spectrum resistance.

## Introduction

The world’s population is growing, and it is expected that by 2050, there will be an additional 2.3 billion people, putting pressure on our agricultural systems [1,2]. With increasing food demand worldwide, it is of the utmost importance to maintain food security and increase productivity to meet these demands. On the other hand, plant diseases are a constant and devastating threat to sustainable crop production worldwide. Amongst the most notorious and economically important pathogens of crop species are plant pathogenic oomycetes.

Plants are “motionless but not defenceless”. They have developed two sophisticated immune systems, that are intertwined, to perceive as well as respond to pathogens [3–7]. The first line of defense employs cell surface pattern-recognition receptors (PRRs) to recognize microbe-associated molecular patterns (MAMPs). This leads to the activation of defenses against invading pathogens, known as pattern-triggered immunity (PTI) [3,8]. The second line of defense uses disease resistance (*R*)-gene products to respond to effector molecules that are secreted by pathogens to establish successful infections and suppress plant immunity [9]. Effectors are recognized by plant intracellular nucleotide binding-site leucine-rich repeat (NLR) proteins, resulting in effector-triggered immunity (ETI) responses [3,10]. Contrary to PTI, ETI is specific, more amplified, faster and leads to constant immune responses, which are revealed by a hypersensitive response (HR) or cell death [11]. Despite these advanced defense and severe selective forces by their host immunity, successful pathogens such as phyto-oomycetes alter their effector repertoire and avoid host resistance. This co-evolutionary arms race between plants and their pathogens reveals the potential of employing pathogen effector proteins to breed for durable resistance in plants [12].

Although crops can be protected from oomycetes through various management strategies including the use of “fungicides” [13,14], this leads to inflated costs of production and adverse environmental effects. In addition, oomycetes may complete several infection cycles a week on a susceptible host under optimal weather conditions, with pathogen control failure leading to rapid epidemics and crop loss. Globally, efforts aimed at sustainable agriculture are geared toward the use of environmentally friendly mechanisms such as protection through crop resistance to improve crop production.

Undisputedly, deployment of *R*-genes is the most effective, environmentally sound, and widely used strategy for providing disease resistance to crop plants. Although this approach has been actively used for over a century, it is unfortunate that some *R-*genes have been overpowered in a single season due to the evolution of new virulence traits within pathogen populations (resistance-breaking strains) [15,16]. Stacking multiple *R*-genes in one genotype is a promising strategy for breeding more stable and durable resistance [17,18]. Nonetheless, this is a very long and tedious process, hence most current agro-ecosystems lack this *R*-gene assortment to improve the durability of resistance genes. In order to effectively control plant diseases, new strategies and techniques in terms of *R*-gene identification, introgression, functional characterization, and field deployment are needed [19]. One of the strategies is to identify *R-*gene products that can recognize effectors that are highly conserved among strains of a pathogen, “core” effectors. Although the concept of “core” effectors has been well documented in bacteria [20–22] and fungi [23–27], it is yet to be formally described for oomycetes (Table 1). Therefore, this review explores the concept of highly conserved effectors, referred to here as “core” RxLR effectors (CREs) in oomycetes, their role in virulence as well as their potential application in breeding for durable resistance.

**Table 1.** “Core” effectors in different groups of plant pathogens

Group	Organism	Identified core effectors	Virulence role	Reference
Oomycete	*Plasmopara halstedii*	354 [30]	Suppress pattern-triggered immunity and some induce hypersensitive responses	[78]
	*Hyaloperonospora arabidopsidis*	18 [4]	RXLR29 was shown to suppress pathogen-induced callose deposition	[153]
Bacteria	*Ralstonia solanacearum*	60–75 [32]	-	[154–156]
	*Xanthomonas arboricola*	57 [11]	-	[157]
Fungi	*Ustilaginoidea virens*	193 [18]	UV_1261 suppress host plant hypersensitive responses	[158–160]
	*Zymoseptoria tritici*	591 [153]	-	[161]
	*Ustilago maydis*	467 (202)	Pep1 inhibits the activity of the apoplastic maize peroxidase POX12 Cce1 hypothesized to inhibit early plant defense responses in the apoplast Rsp3 has a conserved virulence role of protecting the fungal hyphae from maize antifungal proteins activity. Sta1 a novel core effector with virulence role through host cell-wall modification for disease progression	[27,162–167]
	*Colletotrichum orbiculare*		necrosis-inducing secreted protein 1 (NIS1), targets conserved immune kinases hence interfering with PTI signaling	[115]

## Oomycetes RxLR effectors

Oomycetes comprise a group of successful filamentous microorganisms that threaten not only global food security but also natural ecosystems [28,29]. Most notorious among oomycete species is the hemibitrophic genus *Phytophthora*, also known as “the plant destroyers” [30–33]. Another group of plant devastating oomycete species is the obligate biotrophs including downy mildews, *Bremia lactucae* and *Plasmopara viticola* [34,35]. The success of these pathogens is attributed to their ability to secrete an arsenal of effectors. Oomycete genomes encode both extracellular (apoplastic) and intracellular (cytoplasmic) effectors [29]. Apoplastic effectors comprise of cell-wall degrading enzymes [36,37], elicitins [38], and protease inhibitors [39,40]. Contrary to apoplastic effectors that are secreted by the pathogen and execute their pathogenic activity outside of the host cell, cytoplasmic effectors are secreted and translocated into host cells [41,42]. To date, Arginine-any amino acid-Leucine-Arginine (RxLR), Crinkler (CRN) and cysteine, histidine, x, cycteine (CHXC) are the three classes of oomycete cytoplasmic effectors that have been identified [29,31,43].

RxLR-containing effectors represent a rapidly evolving class of effectors that are associated with the biotrophic phase of oomycetes infection [43]. This could be true since most of these effectors have been shown to be highly expressed at the early infection stage and are required for suppression of host immunity [44–49]. In addition, necrotrophic oomycetes, including *Pythium* species were previously thought to be lacking any RxLR-encoding genes [50–52]. Yet, Ai, Yang [53] predicted a total of 359 putative RxLR effectors from nine *Pythium* species. Therefore, it is possible that RxLR effectors in oomycetes share a common ancestor.

Owing to the tremendous advancements in next-generation sequencing technologies, several genomes of phytopathogenic oomycetes have been sequenced [29,31,35,54,55]. This allows a detailed analysis of existing trench-warfare scenario between pathogens through secretion of effectors and host plant-elicited defenses. To date, there are several reviews on the role of RxLR effectors in pathogen–host interaction [56–62]. We can now comprehend the high level of diversity among oomycete effectors, and similarly, their conservation within and among species also known as “core” effectors”. For us to have a better understanding on how “core” RxLR effectors can be utilized in breeding for durable resistance, it is crucial to answer the following questions: What are “core” effectors? Do the available sequenced genomes of oomycetes encode “core” RxLR effectors (CREs)? Do these CREs play a crucial role in virulence activity? Can these CREs be harnessed for durable resistance breeding? What is the future of CREs?

## What are “core” effectors?

Operationally, a core can be defined as a set of all genes shared as orthologs by all members of an evolutionarily coherent group [63]. In the context of effector genes from phytopathogens, the term emerged from high-throughput genomic sequencing study of cassava bacterial pathogen, *Xanthomonas axonopodis* pv. *manihotis* [21]. The study reported a set of conserved effectors (core effectors) that were preserved over three continents, 11 different countries, and seven decades of evolution. This gave birth to a vague definition of “core” effectors as effector proteins that are widely distributed across a population of a particular pathogen [22]. Since effector genes are crucial in pathogen virulence [64], a *bona fide* core effector must be [1]: highly conserved among diverse strains [2], highly expressed during infection, and [3] indispensable for virulence activity. Based on existing studies, it is evident that several conserved RxLR effectors of oomycetes have been identified however, only a few of these have been functionally characterized. Thus, in this review, we define “core” RxLR effectors as those that are either conserved among strains of a pathogen or different pathogen species, with the potential of playing a virulence role during infection process as well as those that have been validated to play a role in virulence. We argue that a pathogen cannot afford to lose “core” effectors since they are indispensable for virulence activity. Therefore, in the absence of functional redundancy, “core” effectors can be key drivers in search for durable resistance considering that; despite different selection pressures coming from diverse hosts and ecosystems, these effectors lack the freedom to mutate. This could probably be due to their location in the gene-dense/repeat-poor regions of the genome [65,66].

## Do genomes of oomycetes encode “core” RxLR effectors (CREs)?

Over the last decade, the genomes of over 65 oomycete species have been sequenced [29,67]. Analyses of these genomes revealed that oomycete genome sizes vary from 32.1 to 295.3 Mb in *Peronospora effuse* and *Plasmopara obducens*, respectively [29,35]. In addition, RxLR secretomes of oomycetes vary significantly. For instance, predictions of RxLR effector genes in the genomes of *P. multivora, P. infestans, P. palmivora,* and *P. megakarya* encode 84, 500–563, 991 and 1181 RxLR effectors, respectively [31,68,69]. This effector content variation has been largely attributed to their location in repeat rich gene sparse regions of the genome [31,70,71]. This promotes genome plasticity as well as genetic variation of effector genes. In addition, expansion of RxLR effector family in *Phytophthora* species was suggested to be through gene duplication and rapid divergence, which could have resulted from illegitimate recombinations [72–74].

**Figure 1.**
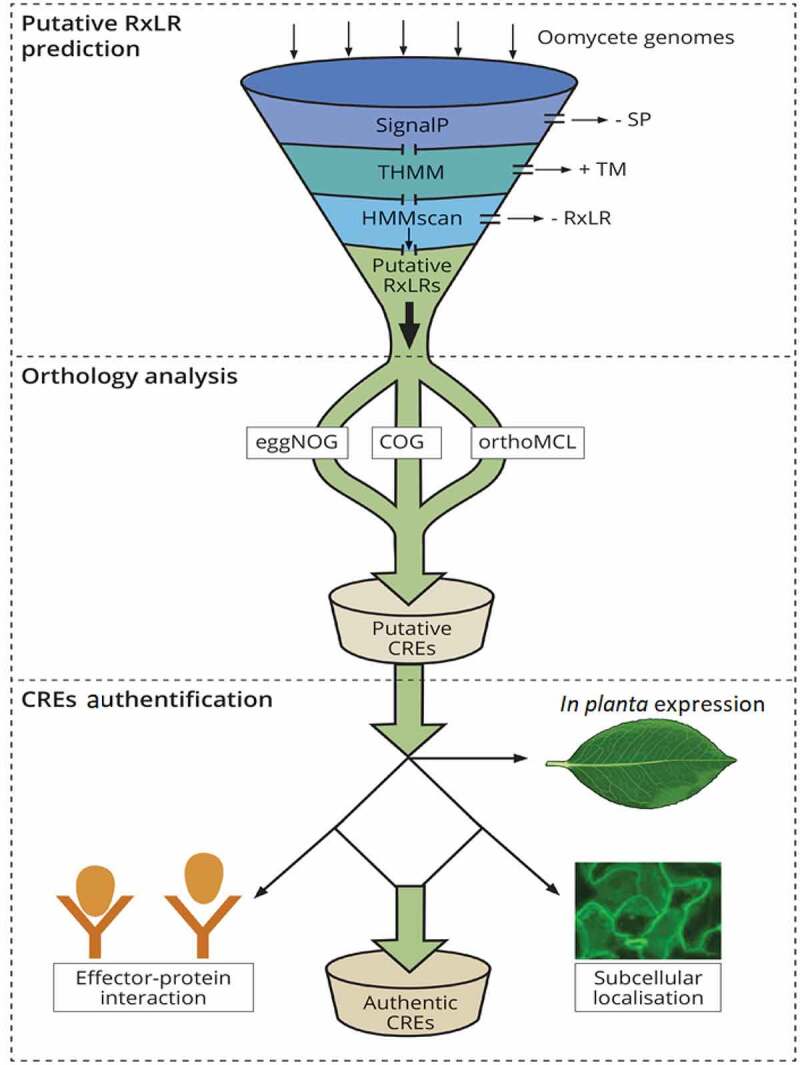
A schematic representation of *in silico* prediction and validation of putative CREs in oomycetes. The secretome prediction pipeline begins with the removal of proteins without a signal peptide (SP) while retaining those with a transmembrane domain (TM), by use of signalP tool and THMM, respectively. Effector proteins with a TM are discarded after signal peptide cleavage as these proteins are not likely to be retained in the plasma membrane. This is followed by removing effector proteins without the signature RxLR motif using HMMscan tool. Orthology analysis is performed to determine RxLR effectors that are conserved within strains or within species of a pathogen (CREs) using orthology analyses tools like COG, eggNOG or orthofinder. The final output is composed of putative secreted CREs with a SP, RxLR motif and without a TM. This output is further authenticated through *in planta* expression to ascertain their role in virulence for instance, their role in enhancing/suppressing host immunity, localization *in planta* using confocal microscopy as well as interacting proteins within host partners

**Figure 2.**
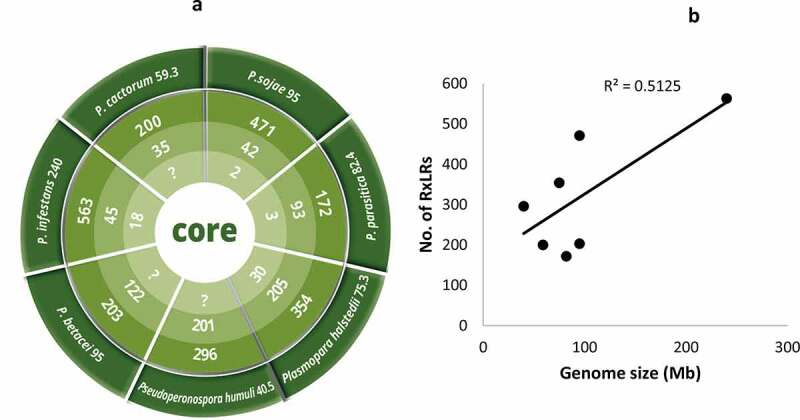
Illustration of phytopathogenic oomycetes with their respective genome sizes (Mb) on the outermost ring. Most of the genomes are *Phytophthora spp* (p). In terms of genome size, *P. infestans* and *Pseudoperonospora humulis* recorded the highest (240Mb) and lowest (40.5Mb) genome sizes, respectively. Counting from the outside, the second ring is the total number of predicted RxLR effectors ranging from 172 in *P. parasitica* to 563 in *P. infestans*. The third ring is the total number of putative CREs while the fourth ring is the total number of authentic CREs with *Plasmopara halstedii* recording a total of 30 CREs. In terms of association between genome size and the number of predicted RxLR effectors in oomycetes, insignificant positive correlation (P = 0,07;R^2^ = 0.51, at 95% confidence level) was recorded (b)

Despite the gain and loss of RxLR effectors in oomycetes due to various selection pressures from plant hosts as well as ecosystems, a small number of these are conserved across the population of a particular species and/or the genus. To date, a considerable number of CREs have been identified. This milestone is attributed to the availability of sequenced genomes of various species of oomycetes. In addition, the presence of N-terminal signature motifs mainly the RxLR-ERR and signal peptide [75] has enabled the identification of various CREs using *in silico* bioinformatics-based approaches. These approaches allow large-scale identification of oomycete RxLR effector arsenals [76–79]. A typical pipeline used in mining CREs in oomycete species is illustrated in Figure 1 leading to identification of putative CREs in some oomycete species (Figure 2). A general approach of the pipeline begins with mining of the genome for effectors by determining their ability to be secreted (presence of a signal peptide and lack of transmembrane domains) and finally, authentication of these effectors through *in planta* expression patterns as depicted in Figure 1.

Besides bioinformatics prediction of putative CREs, genome comparisons can be employed to identify these effectors [80]. For instance, comparing genomes of strains of a species can aid in the identification of sequence polymorphisms, particularly single nucleotide polymorphisms (SNPs) in the protein-coding regions of effectors [64,81]. Generally, effectors are under strong selection pressure, and they are typified by high dN/dS (ratio of non-synonymous and synonymous substitutions) (Bos et al., 2009). However, conserved effectors are well-marked with low ratios of substitutions that change amino acids (non-synonymous) to substitutions that do not change amino acids (synonymous) coupled with significantly low or no copy number variation amongst effector genes, while the opposite applies to non-conserved RxLR effectors [64,82]. This is the case since conserved effectors are believed to be ancestral and because of their obligate role in pathogen virulence, they are subject to purifying selection [83]. This clearly demonstrates how important “core” effectors are since mutations could be detrimental to fitness and subsequently have low fixation probabilities.

Genomic comparison of the potato late blight pathogen *P. infestans* identified about 563 RxLR effectors, with 45 of these being shared among the three strains (06_3924A, NL07434, and T30-4) and expressed *in planta* as CREs [64]. Among these 45 CREs, five *Avr* genes (*Avr2, Avr3a, Avrblb1, Avrblb2,* and *Avrvnt1*) are known gain-of-virulence variants [19]. It was further revealed that *Avr2* and *Avr3a* contain sequence polymorphisms that potentially enable them to evade recognition by cognate *R*–gene products in plants. Likewise, CREs *Avrblb1, Avrblb2,* and *Avrvnt1* have intact coding sequences that are induced during infection [64]. These three *Avr* effectors are therefore predicted to be recognized by their cognate immunoreceptors. Yin, Gu [46] employed next-generation transcriptome deep sequencing strategy coupled with sequence polymorphisms to identify 18 candidate CREs in *P. infestans*. A recent study on genome re-sequencing of *P. sojae* identified a set of 471 RxLR effectors across 26 genomes with 42 of these being conserved as well as expressed *in planta*. Among the 42 “core” effectors, two RxLR effectors, PsAvh241 and PsAvh23 have been demonstrated as essential for full virulence of *P. sojae* [84,85]. This insinuates that the remaining 40 effectors could be critical in the infection process.

To further characterize the level of allelic diversity in RxLR effectors of the oomycete *Phytophthora*, pathogen enrichment sequencing (PenSeq) method has been devised [86]. The method enables the identification of either the presence or absence of variations as well as sequence polymorphisms in important genes of a pathogen, which is a criterion for the effective deployment of host resistance genes [86]. At this point, it is evident that bioinformatics-based approaches have been successfully used in CREs identification. However, in this success, therein lies a trap. With this approach, effector proteins lacking a signal peptide (SP) are discarded. However, these SP-lacking RxLR effector proteins have been shown to be secreted unconventionally [87]. Specifically, RxLR candidate effectors of *P. infestans* were detected in pelleted samples of culture filtrates, providing compelling evidence that these effectors could be delivered using the extracellular vesicles (EVs) [88]. Therefore, most CREs could be overlooked when using bioinformatics approaches only. In addition, bioinformatic identification of RxLR effectors in phytopathogen oomycetes largely depends on the signature motif RxLR. Nonetheless, this motif has been found to be degenerate [89,90]. To circumvent this enigma, EffectorO pipeline was recently developed [91]. The pipeline can predict novel effectors in oomycete genomes independent of motif-based searches. This approach is intended to expand the candidate effector repertoire of narrow host range oomycete plant pathogens.

Mass spectrometry is a powerful technique that can be used to solve deficiencies of *in silico* prediction of RxLR effectors. It has been previously employed in validating computationally predicted RxLR effector proteins to be secreted as well as identifying extracellular proteins that lack typical SP, which would then be overlooked [92–94].

Taken together, coupling *in silico*-based approaches with experimental techniques such as mass spectrometry could be the gold standard for identifying CREs and more so, novel CREs that have no matches in public databases. In addition, there is a need to verify whether indeed RxLR effectors form part of the cargo that is being delivered to the extracellular environment of the pathogen using EVs. More importantly, the association of RxLR effectors with EVs during their biogenesis is worth investigating.

## Do “core” RxLR effectors (CREs) play a role in virulence?

Despite the presence of a multi-layered immune response in plants [7], CREs subdue host immune responses by targeting key components leading to disease proliferation. In most cases, these targeted components are central cellular processes/proteins that are conserved across diverse plant species. The last two decades have witnessed the identification of conserved RxLR *Avr3a*, in *P. infestans* with its cognate *R*-gene in the host cell [95]. Subsequent characterization of this effector revealed its crucial role in preventing host cell death during the biotrophic phase of infection by interacting with and stabilizing the host ubiquitin E3-ligase CMPG1 [96]. Since then, several other studies have been carried out. For instance, *P. sojae* RxLR effector, PsPSR2, that is conserved among eight *Phytophthora spp*, suppresses RNA silencing activity in various plants [97], an activity that has been reported in novel RxLR effectors [98]. Another *P. sojae* RxLR effector (PsAvh73) homologous to oomycete *Hyaloperonospora arabidopsidis* effector (HaRxL23) was reported to suppress PTI responses in *Nicotiana benthamiana* and ETI in *Glycine max* [48]. *Phytophthora brassicae* effector, RxLR24, was showed to be highly conserved among most successful species of *Phytophthora* such as *P. infestans, P. sojae*, and *P. parasitica var. nicotianae* [99]. Further, characterization of this effector and its close homolog in *P. infestans*, PiRxLR24, revealed that the two effectors localize to the plasma and vesicular membranes, where they associate with members of the RABA GTPase subfamily, hence interfering with vesicle tracking of the host plant [99]. In a separate study, three “core” effectors of *P. parasitica*, PpRxLR2, PpRxLR3, and PpRxLR5 were highly expressed in *N. benthamiana* leaves during infection [76]. Further analysis showed that effector PpRxLR2 enhanced the virulence of *P. parasitica* via complete suppression of the INF-1 induced PCD, while effectors PpRxLR3 and PpRxLR5 partially suppressed host plant defenses. Two CREs, REX3 and REX2, of the broad host-range oomycete *P. palmivora* were demonstrated to promote disease development upon expression, where effector REX3 enhanced virulence of the pathogen by interfering with host secretion pathways [100]. The well-studied oomycete *P. infestans* was reported to harbor a total of 18 CREs that are not only expressed during the early phase of infection, but also contribute to disease development by inhibiting plant defense responses induced by both PTI and ETI [46]. Although CREs are known to target positive regulators of host immunity, it is fascinating that they also target negative regulators of host immunity called susceptibility factors (SFs). A good example is Avr3a-related RxLR effectors that are distributed across diverse *Phytophthora* species. These effectors target the family of cinnamyl alcohol dehydrogenase 7 (CAD7) leading to downstream suppression of PTI [101].

We believe that evolutionary conservation is good since motif occurrences that are unlikely to have functional importance are eliminated hence retaining those motifs/domains that are crucial for the pathogen to successfully infect the host. Although CREs are said to be highly conserved, their essentiality is not likely to be retained through the conservation of overall proteins but through specific protein domains. This is driven by the specificity of substrate recognition, and it is therefore anticipated that active site residues are preserved better compared to the overall protein conservation. From the few existing studies on CREs of oomycetes, there is no specific domain that has been implicated in effector virulence activity [97,99,101]. Interestingly, alignment analyses of these CREs reveal the presence of C-terminal W (Trp) and Y (Tyr) motifs [102,103]. We therefore hypothesize that these motifs could be crucial in virulence activity of CREs of oomycetes since these motifs have been implicated in effector function [96,102,104,105]. Studies have shown that approximately 44% of *Phytophthora* RxLR effectors and 26% of *H. arabidopsidis* possess a highly conserved W and Y motif at the C-terminal [102,103]. Structural analyses of WY motif(s) have revealed the presence of more than one α-helix bundle formed by each motif [106]. It is hypothesized that the α-helical-domain, which is the “WY-domain”, enhances effector adaptation through mutations, while the hydrophobic core fold provides stability and flexibility therefore, implicated in virulence activities of the effector [103]. Following this hypothesis, studies have reported that this hydrophobic core is crucial in effector-host target protein interaction [105,107,108], cell-death induction [109,110] RNA silencing suppression activity and suppression of PTI and ETI events [48],101.

Although the WY motifs have been associated with effector virulence activity, *P. infestans* RxLR effector PexRD54, was shown to have a total of five WY repeats but surprisingly, the virulence activity of the effector was dependent on a C-terminal ATG8-interaction motif (AIM) that binds proteins related to autophagy (ATG8) [111,112]. Further analysis of PexRD54 revealed that the AIM motif, at the C-terminus of the effector, is linked to the last WY domain by a short helix [112]. Therefore, we can hypothesize that the main function of WY motifs in RxLR effectors is to act as a “dais” to introduce functional motifs or domains for interaction with host plant proteins. Recently, the highly conserved Avr3a-like effectors from *Phytophthora* species showed a conserved function by targeting plant CAD7 subfamily [101]. Amazingly, this function was independent of a putative enzyme active site of these effectors. Since the sequence conservation of these proteins revealed the presence of conserved WY motif at the C-terminal, we can therefore propose that WY motif could be responsible for Avr3a-like effectors-CAD7 interaction.

Although it appears that W-Y motifs are crucial in virulence activity of most RxLR effectors in oomycetes, it is not apparent whether these motifs are key players in CREs activity. Therefore, dissecting the structure of CREs using experimental and computational approaches is encouraged. This will inform not only the functional motifs or domains but also the host immune proteins or processes that these CREs target.

## Do CREs target “core” host proteins/processes?

Since “core” effectors are maintained in effector repertories over a long evolutionary time [66], they are likely to target conserved elements in the plant immune system or metabolism that facilitate host colonization [113,114]. It is also important to know that these targeted host proteins and processes are “the candy liked by many” since they are crucial processes that cannot be altered or eliminated without complete damage to plant fitness. The concept of “core” effector targeting a conserved host protein has been witnessed in plant fungal effectors [27,115] and also in effectors (AvrB, AvrPto, HopAI1) of bacteria such as *Pseudomonas syringae* [116–118]. In oomycetes, this concept has not been sufficiently exploited. A recent study revealed that RxLR effectors target various plant processes with vesicle trafficking being a major targeted process [119]. A few studies have explored whether RxLR effector proteins target conserved process. For instance, evolutionarily conserved RxLR effectors in oomycetes *H. arabidopsidis* and *P. sojae* were shown to suppress immunity in plant species that are divergent from the source pathogen’s host [48,81]. In the same token, several conserved RxLR effectors from oomycete *P. agathidicida*, a pathogen of gymnosperms, were revealed to interact with the immune system of model angiosperm plants (*Nicotiana spp*), in a similar way to that of angiosperm pathogens [120]. These findings provide a hint of possible interaction of conserved effectors with conserved host targets. Recently, Avr3a-like conserved effectors from *Phytophthora* pathogens were reported to target a negative regulator of immunity, CAD7 in both *Arabidopsis thaliana* and *N. benthamiana* leading to disease development [101]. The notion of oomycetes’ effectors targeting negative regulators/susceptibility factors is currently a fertile ground for potential “core” effectors as reviewed by [121].

At this point in time, we cannot confidently conclude that CREs of oomycetes target broadly conserved plant proteins, nonetheless, the presence of “core” effectors in these pathogens could explain the success of most broad host-range oomycetes, notably *Phytophthora* species. To fully understand this concept, functional characterization of “core” effectors is key. This can be achieved through screening for protein–protein interactions using a yeast two-hybrid system (Y2H) [122,123], followed by validation of the interaction through co-immunoprecipitation [124]. Other validation methods include biotinylation [125] and bimolecular fluorescence complementation (BiFC) [126,127]. Further, mutation analyses like site-directed mutagenesis [128,129] and virus induced gene silencing, VIGs [130,131] can be performed to gain more insight on effector-host protein interaction.

## Can “core” effectors be useful in breeding for durable resistance?

Currently, there is a paradigm shift from conventional to breeding for durable resistance. Effectoromics is a high-throughput functional genomics approach that employs the use of effectors to probe plant germplasm [132,133]. Here, we reason that since “core” effectors are present in most strains or species of a pathogen as well as playing an important role in virulence, a pathogen cannot easily lose them even after a new resistance gene is deployed in the host. Consequently, *R-*gene products that recognize such effectors are anticipated to be more durable than resistance gene products that perceive non conserved effectors.

The journey to durable resistance using “core” effectors starts with employing next-generation sequencing technologies to sequence and assemble genomes of various pathogens that are responsible for disease in different fields. Using computational approaches, “core” effectors in these strains can be identified. Consequently, these “core” effectors can be employed as probes in screening for cognate *R* proteins from wild germplasm using mainly transient co-expression assays [134] followed by either marker-assisted breeding or transgene deployment [12,22,135]. Validation of these new *R-*genes could be enhanced by new genome-editing methods like clustered regulatory interspaced short palindromic repeat (CRISPR) technologies [136].

One fascinating fact about RxLR effectors is their ability to operate as “double edge swords”, where on one side they suppress host immune responses, while on the other side they act as avirulence (*Avr*) factors leading to R protein mediated defenses in plants [11]. Screening for potential R proteins that recognize Avr RxLR effectors of oomycetes has been attempted [95,137–141]. Nonetheless, efforts have been directed toward the well-conserved *P. infestans* RxLR effector Avr3a [95,142]. This effector exists in two alleles (*Avr3a^KI^* and *Avr3a^EM^*), and this translates to a difference of two amino acids in the mature protein where AVR3a^KI^ is recognized by R3a while AVR3a^EM^ evades R3a recognition [95]. The study marked *Avr3a* as a potential candidate in breeding for durable resistance, however, there was a need to produce potato plants with an enhanced resistance spectrum and durability by integrating naturally occurring *R-*genes or engineered, synthetic *R-*genes with extended pathogen recognition precisions that comprises Avr3a^EM^ recognition. Nine years later, a random mutagenesis study was conducted to generate a mutant version of R3a that recognized AVR3a^EM^ [143]. Intriguingly, mutation of *I2* gene, a close homologue of the *R3a* gene in tomato, made the gene product more responsive to AVR3a hence conferring resistance not only to *P. infestans* but also to the fungal pathogen *Fusarium oxysporum* [144].

Although “core” effectors appear to be the perfect targets in breeding for durable resistance, some studies have reported that due to the long evolution of plant–pathogen interaction, there is a possibility of complex mechanisms coming into place to shield conserved effectors from recognition [145–147]. For instance, the virulence activity of the conserved pathogen-secreted xyloglucan-specific endoglucanase (PsXEG1), an apoplastic effector of *P. sojae*, was shown to be protected by its paralog that is enzymatically inactive by binding more tightly to the host apoplastic glucanase inhibitor GmGIP1 than PsXEG1 [40]. Whether this is also the case in intracellular effectors like those with RxLR motifs needs to be investigated. We therefore suggest that deploying multiple, stacked *R*-genes that recognize “core” effectors can be of importance in reducing chances of a pathogen to overcome resistance.

Another potential way of exploiting CREs for durable resistance and broad spectrum breeding is capitalizing on their ability to target susceptibility (*S*)-genes [101]. Although resistance and susceptibility appear to be opposite sides of the same coin, the two have “resistance” as the focal point. *S-*genes are recessively inherited, with resistance being achieved through the loss of function of a host factor required by the pathogen. On the other hand, *R-*genes are dominantly inherited, and resistance is triggered when a pathogen-derived avirulence determinant is recognized by the R protein [148]. *S-*genes come in two “flavours”: Those that are independent of immunity as they directly serve to promote disease (genuine *S-*genes) and those that promote disease indirectly also termed as negative regulators of immunity [149]. Pathogens may indirectly benefit from the activity of *S*-gene products or directly by forcing plants to cooperate by activating or stabilizing *S* genes or their products, with the help of effectors [148]. A review by He, McLellan [121] documents several RxLR effectors of plant pathogenic oomycetes that target host *S-*genes and among these are “core” RxLR effectors [101,123]. We therefore reason that durable as well as broad-spectrum resistance can be attained by identifying those susceptibility genes that are targeted by CREs, using protein–protein interaction methods such as yeast-two hybrid screening [96,123]. After the identification, inactivation of these *S-*genes by mutations or genome editing [150] is performed with the aim of interfering with the ability of the effectors to associate with their host partners [148,151]. For instance, potato plants showed complete resistance to *P. infestans* after successful knockdown of six *S-*genes [152]. Targeting *S*-genes seems to be an avenue to breeding for durable resistance using “core” effectors, however, there is a cause for alarm since introduction of mutations to susceptibility genes has been linked to pleiotropic effects, specifically dwarfism and sensitivity to stress [148,151]. This limits the utilization of these genes in agriculture. Therefore, for an *S*-gene mutant to be practical in crop breeding, the following questions should be considered: (i) Does mutation or editing of an *S-*gene have undesirable side effects? (ii) In a scenario where an *S*-gene is redundant, is it possible to target multiple genes? (iii) Will targeting an *S-*gene for mutation result in sufficiently improved resistance?

## What is the future of CREs research?

There is a clear potential for “core” effectors to target conserved processes in diverse host plants [115]. However, studies on the ability of CREs in oomycetes to target conserved host processes/protein have not been fully explored. Therefore, functional studies on these effectors are highly encouraged. The emerging reports that genomes of oomycetes species encode CREs shed light on important virulence roles played by these effectors [46,48,81,99]. Nevertheless, some key questions remain to be answered: Why do oomycetes conserve some RxLR effectors? Do these effectors play conserved roles in targeting host plant defenses? Do these effectors act as probes in screening for cognate *R-* genes in search for durable resistance in plants? Providing answers to these questions can potentially further advance the field. In addition, to gain further insight of the biology of “core” effectors in oomycetes, biochemical, genetic as well as biophysical studies are highly encouraged.

Although less has been documented on “core” RxLR effectors in oomycetes, the few existing studies have identified putative CREs through *in silico* prediction-based approaches. The task ahead is to validate the expression of these effectors *in planta* to have consensus in defining the term “core effectors”. In addition, functional characterization is worth undertaking to dissect these effectors and hence identifying specific domains that are conserved as well as important in virulence roles of these effectors. Therefore, harnessing CREs as future breeding tools to increase host resistance requires extensive collaborations between plant breeders, geneticists, and phythologists.

*Figures in and outside the parenthesis are the “core” and potentially secreted effectors respectively*

## Data Availability

The authors confirm that the data supporting the findings of this study are available within the article and freely available, under a license allowing re-use by any third party for any lawful purpose. Data shall be findable and fully accessible.
